# Photocatalytic Activity of the Blends Based on TiO_2_ Nanoparticles and Reduced Graphene Oxide for Degradation of Acetaminophen

**DOI:** 10.3390/molecules28114546

**Published:** 2023-06-04

**Authors:** Monica Daescu, Madalina Chivu, Elena Matei, Catalin Negrila, Oana Cramariuc, Mihaela Baibarac

**Affiliations:** 1 National Institute of Materials Physics, Atomistilor Street 405A, POB MG 7, 077125 Bucharest, Romania; monica.daescu@infim.ro (M.D.); madalina.chivu@infim.ro (M.C.); elena.matei@infim.ro (E.M.); catalin.negrila@infim.ro (C.N.); 2 Faculty of Chemical Engineering and Biotechnologies, 1-7 Gheorghe Polizu Str., Sector 1, 077125 Bucharest, Romania; 3 IT Centre for Science and Technology, 25 no. Av. Radu Beller Str., 011702 Bucharest, Romania; oanacramariuc@yahoo.com

**Keywords:** titanium dioxide, reduced graphene oxide, acetaminophen, photocatalytic properties

## Abstract

The aim of this work is to highlight the influence of blends based on TiO_2_ nanoparticles and reduced graphene oxide (RGO) on the photodegradation of acetaminophen (AC). To this end, the catalysts of TiO_2_/RGO blends with RGO sheet concentrations equal 5, 10, and 20 wt. % were prepared by the solid-state interaction of the two constituents. The preferential adsorption of TiO_2_ particles onto the RGO sheets’ surfaces via the water molecules on the TiO_2_ particle surface was demonstrated by FTIR spectroscopy. This adsorption process induced an increase in the disordered state of the RGO sheets in the presence of the TiO_2_ particles, as highlighted by Raman scattering and scanning electron microscopy (SEM). The novelty of this work lies in the demonstration that TiO_2_/RGO mixtures, obtained by the solid-phase interaction of the two constituents, allow an acetaminophen removal of up to 95.18% after 100 min of UV irradiation. This TiO_2_/RGO catalyst induced a higher photodegradation efficiency of AC than TiO_2_ due to the presence of RGO sheets, which acted as a capture agent for the photogenerated electrons of TiO_2,_ hindering the electron–hole recombination. The reaction kinetics of AC aqueous solutions containing TiO_2_/RGO blends followed a complex first-order kinetic model. Another novelty of this work is the demonstration of the ability of PVC membranes modified with Au nanoparticles to act both as filters for the removal of TiO_2_/RGO blends after AC photodegradation and as potential SERS supports, which illustrate the vibrational properties of the reused catalyst. The reuse of the TiO_2_/RGO blends after the first cycle of AC photodegradation indicated their suitable stability during the five cycles of pharmaceutical compound photodegradation.

## 1. Introduction

Pharmaceutical compounds are among the main organic contaminants that pollute marine environments [1]. Acetaminophen (AC) is one of the most widely used drugs in treating migraines [2], osteoarthritis [3], coronavirus disease [4], fever [5], etc. Studies reported in 2017 have indicated the presence of 0.01 mg/L AC in sewage effluent [6]. Among the catalyst most widely used for AC photodegradation are: (a) TiO_2_ microspheres [7], (b) TiO_2_ nanotubes [8], (c) TiO_2_ modified with KAl(SO_4_)_2_ and NaAlO_2_ [9], (d) activated carbon/Ti_x_O_y_ nanoparticles composites [10], (e) TiO_2_–graphite composites [11], (f) TiO_2_ nanotubes/graphene oxide [12], (g) TiO_2_ (P25)–graphene oxide nanocomposite [13], (h) nanocomposite based on TiO_2_ and reduced graphene oxide (RGO) [14,15,16,17], (i) mesoporous TiO_2_ modified with carbon nanotubes [18], etc. For the composites based on TiO_2_ and RGO, the synthesis methods used include: (a) the mixing of the commercial TiO_2_ with GO suspension, followed by photoreduction via irradiation with UV light [15]; (b) the dispersion of GO and TiO_2_ into a solution of ethanol and deionized water, accompanied by the thermal reduction of GO at 130 °C [14]; (c) the hydrothermal method, which involves adding TiO_2_ into a GO–ethanol solution, followed by a reduction at 120 °C [16,19], and (d) the force-spinning method of fibers based on GO and TiO_2_, followed by an annealing treatment at 500 °C [17,20]. The hydrothermal method has been used for the synthesis of the composites of graphene/TiO_2_ nanotubes [21] and graphene/TiO_2_ nanofibers [22]. Other graphene/TiO_2_ composites with core–shell [23] or 3D [24] structures were obtained by sol-gel methods [23] and ball milling accompanied by a hydrothermal method [24], respectively. Depending on the preparation method used for the TiO_2_/RGO composite’s synthesis, the AC degradation efficiency has been reported to be equal to 100% [14] and 92% [16]. The photocatalytic mechanism of the graphene/TiO_2_ composites under irradiation with UV and VIS light, respectively, was discussed in a recent review published by B. Tang et al. [25].

In this paper, the TiO_2_/RGO blends will be prepared by the solid-phase interaction of TiO_2_ particles with RGO sheets. Compared to the synthesis methods reported for TiO_2_- and RGO-based catalysts [11,14,15,16,17,18,19,20,21,22,23,24], the advantages of solid-phase interaction are the small number of reactants and the short samples preparation time that ensures the easy scaling of TiO_2_/RGO blends. In order to show the vibrational changes induced by the solid-phase interaction of the two constituents, the characterization of TiO_2_/RGO blends will be performed by Raman scattering and FTIR spectroscopy. The morphology of TiO_2_ particles and TiO_2_/RGO blends will be analyzed by scanning electron microscopy (SEM). Characterization of the TiO_2_ particles, RGO sheets, and TiO_2_/RGO blends by X-ray photoelectron microscopy will also be carried out. The photocatalytic properties of the TiO_2_/RGO blends in relation to AC photodegradation in the presence of UV light will be shown. In this context, the influence of the RGO sheets’ weight within the TiO_2_/RGO blend, the concentration of the TiO_2_/RGO blends, as well as the presence of excipients on the AC photodegradation will be reported. The effects of TiO_2_/RGO blends on AC degradation as dependent on the AC solution’s concentration as well as in real samples will also be shown.

The separation of a catalyst after the photodegradation of pollutants and its reuse are important topics today [26]. In this work, the ability of PVC membranes containing Au nanoparticles to act both as filters for the removal of TiO_2_/RGO blends after AC photodegradation and as SERS supports to illustrate the chemical adsorption of the TiO_2_/RGO blends from decontaminated waters will be shown. The recovery and reuse of the TiO_2_/RGO blends after five cycles of AC photodegradation, and the analysis of the variations in the degradation efficiency, will also be shown. The results reported in this paper will demonstrate that the solid-phase interaction method allows obtaining TiO_2_/RGO blends with a photodegradation efficiency of AC aqueous solutions equal to 95.18%, having suitable stability while reusing the catalyst in successive photodegradation cycles of this pollutant. To validate the stability of the catalyst, the ability of PVC membranes modified with Au nanoparticles to act both as filters for the removal of TiO_2_/RGO blends after each cycle of AC photodegradation and as potential SERS supports, which to illustrate the vibrational properties of the reused catalyst will be shown.

## 2. Results and Discussion

### 2.1. Optical Properties of TiO_2_/RGO Blends

**Figure 1:**
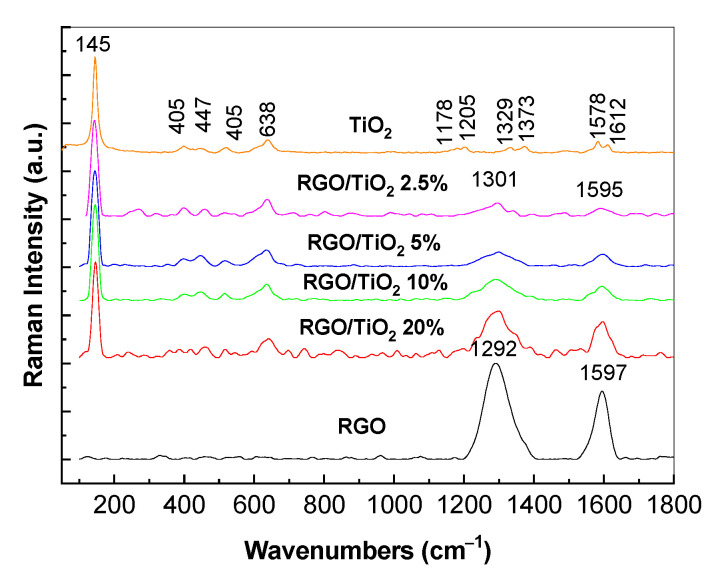
Raman spectra of TiO_2_ particles (orange curve), RGO sheets (black curve), and their blends with concentrations of the RGO sheets equal to 2.5 wt. % (purple curve), 5 wt. % (blue curve), 10 wt. % (green curve), and 20 wt. % (red curve).

Figure 1 shows the main Raman lines of TiO_2_ at 147, 405, 447, 523, and 638 cm^−1^, and 1178, 1205, 1329, 1373, 1583, and 1612 cm^−1^, which have been assigned to the vibrational modes of E_g_ in TiO_2_ anatase (A), B_1g_ in TiO_2_ A, E_g_ in TiO_2_ rutile (R), B_1g_ in TiO_2_ A, Eg in TiO_2_ A, a complex band of the Raman lines at 523 + 638 cm^−1^, the third order of Raman lines at 401 and 447 cm^−1^, the complex band of the Raman lines at 405 + 447 + 523 cm^−1^, the third order of Raman line at 523 cm^−1^ and the presence of water molecules adsorbed onto the TiO_2_ particles’ surfaces [27,28]. In the range of 1000–1700 cm^−1^, the Raman spectrum of RGO shows two bands with peaks at 1292 and 1597 cm^−1^ assigned to the breathing vibrational mode of the hexagonal rings containing carbon atoms and the E_2g_ phonon mode at the center of the Brillouin zone, respectively [29]. As shown in Figure 1, the solid-state interaction of TiO_2_ with RGO induces a cumulative effect of the vibrational modes of the two constituents of the TiO_2_/RGO blends, simultaneously with a shift of the D band from 1292 to 1301 cm^−1^. The shift of the D band indicates an increase in the disordered state in the RGO lattice.

**Figure 2:**
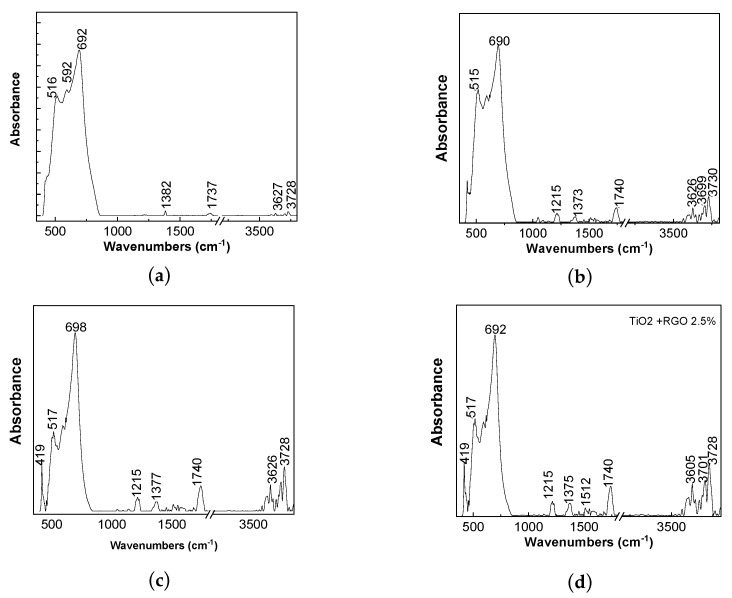
IR spectra of TiO_2_ particles (**a**) and the TiO_2_/RGO blends with concentrations of RGO sheets equal to 5 wt. % (**b**), 10 wt. % (**c**), and 20 wt. % (**d**).

Additional information concerning the solid-state interaction of TiO_2_ with RGO is shown via FTIR spectroscopy in Figure 2. The main IR bands of TiO_2_ are situated in the spectral range of 500–700 cm^−1^, which are accompanied by other IR bands with low absorbance peaks of 1382, 1737, and 3627–3728 cm^−1^. The IR bands peaking at 692, 1737, and 3627–3728 cm^−1^ are assigned to the vibrational modes of stretching of the Ti–O–Ti bond, water molecules, and the stretching vibrational modes of the OH bond [30]. The interaction of TiO_2_ with RGO induces an increase in the absorbance of IR bands localized in the spectral ranges 1000–1800 cm^−1^ and 3626–3730 cm^−1^. The increase in absorbance of the IR bands peaking at 1740 cm^−1^ and 3627–3728 cm^−1^ indicates preferential adsorption onto the RGO sheets’ surface via the water molecules on the TiO_2_ particles’ surfaces.

**Figure 3:**
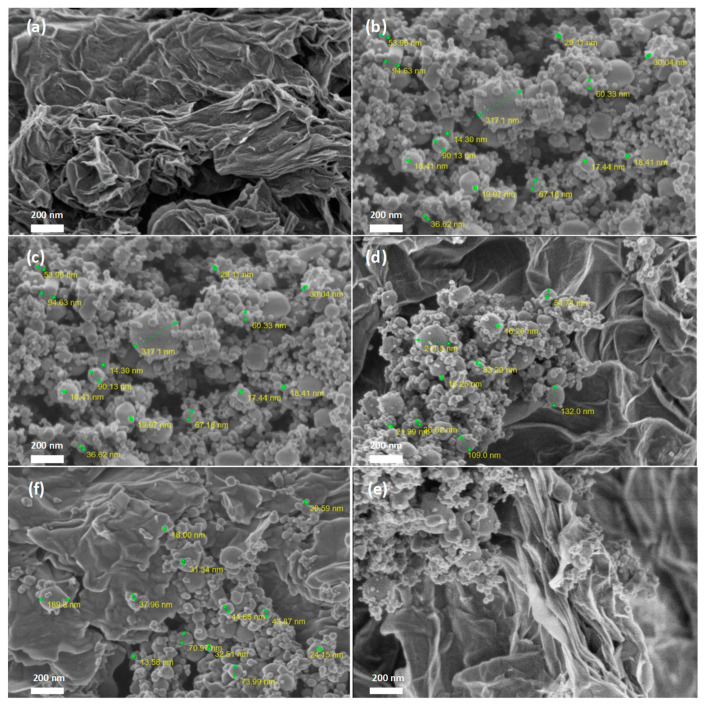
SEM images of the RGO sheets (**a**), TiO_2_ particles (**b**), and the TiO_2_/RGO blends with concentrations of the RGO sheets equal to 5 wt. % (**c**), 10 wt. % (**d**,**e**), and 20 wt. % (**f**).

**Figure 4:**
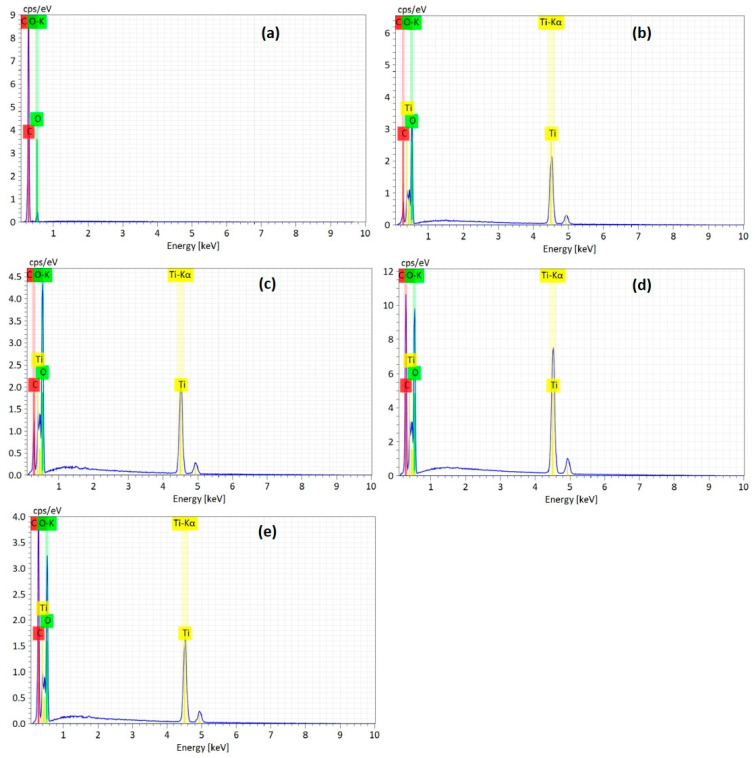
The EDS spectra of the RGO sheets (**a**), TiO_2_ particles (**b**), and the TiO_2_/RGO blends with concentrations of RGO sheets equal to 5 wt. % (**c**), 10 wt. % (**d**), and 20 wt. % (**e**).

Figure 3 and Figure 4 show the morphology of the TiO_2_/RGO blends analyzed by scanning electron microscopy (SEM) and elemental analyses of the samples by energy-dispersive X-ray (EDS).

A careful analysis of Figure 3 indicates: (a) the RGO sheets have many creases (Figure 3a); (b) the TiO_2_ particles’ sizes vary in the 14–317 nm range (Figure 3b); (c) the adsorption of TiO_2_ particles onto the RGO sheets’ surfaces, in the case of TiO_2_/RGO blends with an RGO concentration of 5 wt. % (Figure 3c); (d) the adsorption of the TiO_2_ particles both onto the RGO sheets’ surfaces (Figure 3d) and between the RGO sheets (Figure 3e), when the RGO concentration in the TiO_2_/RGO blends is 10 wt. %; and (e) the presence of a small mass of TiO_2_ particles adsorbed onto the surfaces of the RGO sheets, most of which are covered by the RGO layers (Figure 3f). The presence of Ti, O, and C in the TiO_2_/RGO blends is clear highlighted in Figure 4.

**Figure 5:**
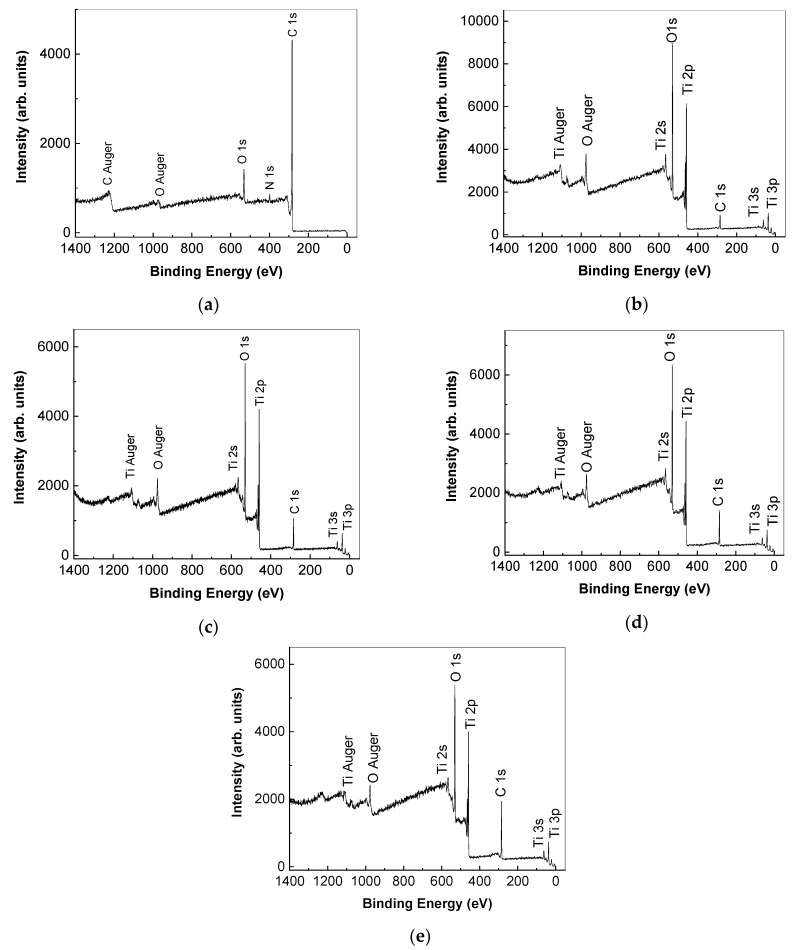
X-ray photoelectron spectroscopy spectra of the RGO sheets (**a**), TiO_2_ particles (**b**), and the TiO_2_/RGO blends with concentrations of RGO sheets equal to 5 wt. % (**c**), 10 wt. % (**d**), and 20 wt. % (**e**).

Figure 5 shows the X-ray photoelectron spectroscopy (XPS) spectra of the RGO sheets, the TiO_2_ particles, and the TiO_2_/RGO blends with concentrations of RGO sheets equal to 5 wt. %, 10 wt. %, and 20 wt. %. Analysis of the XPS spectra indicates the following atomic concentrations: (a) TiO_2_ particles comprise 19.4% C, 57.2% O, and 23.5% Ti; (b) the RGO sheets comprise 89.9% C, 7.7% O, and 2.4% N. The atomic percentages in the TiO_2_/RGO blends are as follows: (a) those with 5 wt. % RGO comprises 30.5% C, 48.8% O, and 20.7% Ti; (b) those with 10 wt. % RGO comprises 33.4% C, 47.7% O, and 19% Ti; (c) those with 20 wt. % RGO comprise 47.1% C, 37.7% O, and 15.2% Ti.

**Figure 6:**
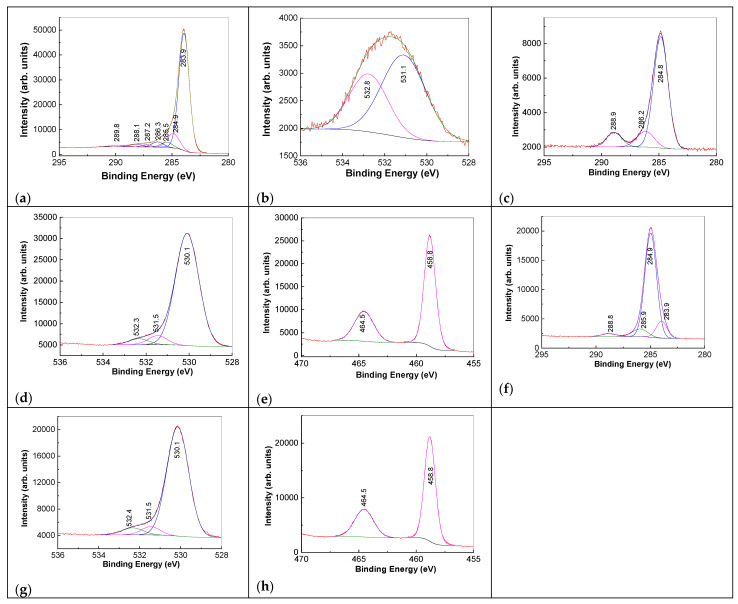
The C1s and O1s XPS spectra of the RGO sheets (**a**,**b**). The C1s, O1s, and Ti2p XPS spectra of TiO_2_ particles (**c**–**e**) and TiO_2_/RGO blends with concentrations of RGO sheets equal to 20 wt. % (**f**–**h**). In all figures, the black curves correspond to the experimental data.

Figure 6a,b shows the deconvolution of the C1s and O1s XPS spectra of RGO sheets. Figure 6a highlights an intense peak at 283.9 eV, with was assigned to the bonds of the type C-C, C=C, and C-H, as well as other six of low intensity peaked at 284.9, 285.5, 286.6, 287.2, 288.1, and 289.8 eV that were assigned to the bonds of the type C-O, C=O, and -COOH [31]. Ti2p XPS spectra of TiO_2_ particles are dominated by an intense peak at 464.5 eV accompanied by another peak localized at 458.8 eV (Figure 6e), which were assigned to the Ti 2p_1/2_ and Ti 2p_3/2_ [6]. In the case of the TiO_2_/RGO blend with a concentration of RGO sheets equal to 20 wt.%, Figure 6f–h is similar to those of C1s, O1s, and Ti2p XPS spectra of TiO_2_, the only difference being observed in the C1s XPS spectrum profile, where there is a significant decrease in the maximum located at 283.9 eV.

Considering this information regarding the TiO_2_/RGO blends, in the following, the photocatalytic properties of these materials in relation to the photodegradation of AC will be shown.

### 2.2. Photocatalytic Properties of the TiO_2_/RGO Blends

The photodegrading efficiency of AC in the presence of catalysts comprising TiO_2_ and TiO_2_/RGO blends can be calculated with the following equation:

$$D_{\mathrm{eff}}=\left(\frac{A_{0}-A_{t}}{A_{0}}\right)\times 100$$

where A_0_ and A_t_ are the values of the absorbance of the band peaking at 320–338 nm, corresponding to exposure to UV light for 0 min and 100 min, respectively. Before each measurement, the UV-VIS spectra of samples under dark conditions for 30 min were recorded in order to establish the adsorption/desorption equilibrium of AC in relation to the catalysts.

**Figure 7:**
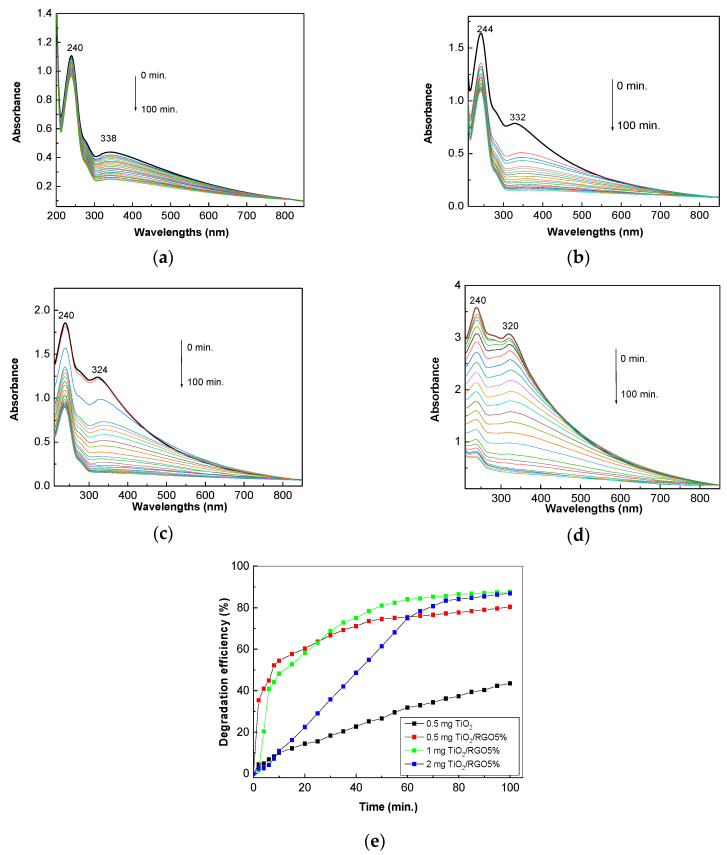
Evolution of the UV-VIS spectra of the AC in the presence of 0.05 mg/mL TiO_2_ (**a**) and the TiO_2_/RGO blends with RGO concentrations of 5 wt. % during exposure to UV light for 100 min. The weights of the TiO_2_/RGO composite added to the 0.2 mM AC solution were: 0.05 mg/mL (**b**), 0.1 mg/mL (**c**), and 0.2 mg/mL (**d**). Degradation efficiency of AC in the presence of 0.05 mg/mL TiO_2_ and various weights of TiO_2_/RGO blends with an RGO concentration of 5 wt. %, i.e., 0.5 mg, 1 mg, and 2 mg, which were dispersed each in 10 mL AC aqueous solution 0.2 mM, after subsequent exposure to UV light (**e**).

Figure 7 shows UV-VIS spectra of the aqueous solution of AC 0.2 mM in the presence of 0.05 mg/mL TiO_2_ and 0.05 mg/mL, 0.1 mg/mL, and 0.2 mg/mL of TiO_2_/RGO blends with RGO sheet concentrations equal to 5 wt. %.

According to Figure 7a, the UV-VIS spectrum of the TiO_2_ dispersed in the AC aqueous solution shows two bands peaking at 240 nm and 338 nm, which have been assigned to electronic transitions of the types n–π* [32] and π–π* [33], respectively. Before exposure to UV light, increasing the concentration of TiO_2_/RGO blend in the AC aqueous solution from 0 mg/mL to 0.05 mg/mL, 0.1 mg/mL, and 0.2 mg/mL induced: (i) a blue shift of the band assigned to the π–π* electronic transition of the C=O bond in the amide group from 338 nm (Figure 7a) to 332 nm (Figure 7b), 324 nm (Figure 7c), and 320 nm (Figure 7d); and (ii) a change in the ratio between the absorbance of the bands peaking at 240–244 nm and 338–320 nm from 2.53 (Figure 7a) to 2.09 (Figure 7b), 1.5 (Figure 7c), and 1.16 (Figure 7d). The exposure of the sample of TiO_2_ dispersed in an AC aqueous solution to UV light leads to a gradual decrease in the absorbance of bands, as observed in Figure 7. The efficiency of AC photodegradation in the presence of TiO_2_ is 43.5%. The addition of RGO sheets to TiO_2_ in order to prepare TiO_2_/RGO blends with RGO concentrations of 5 wt. % leads to an increase in the efficiency of AC photodegradation. Thus, in the presence of 0.05 mg/mL, 0.1 mg/mL, and 0.2 mg/mL of TiO_2_/RGO blend with the RGO concentration of 5 wt.%, the AC photodegradation efficiency is equal to 80.41%, 87.64%, and 86.95% (Figure 7e). The greater efficiency in the case of the TiO_2_/RGO blends compared to TiO_2_ can be explained by the fact that RGO has conductive properties that reduce the electronic accumulation on the surfaces of TiO_2_ particles, thus leading to a reduction in the recombination of the electron–hole pair. This results in an increase in the photocatalytic properties of TiO_2_ in the presence of RGO sheets.

**Figure 8:**
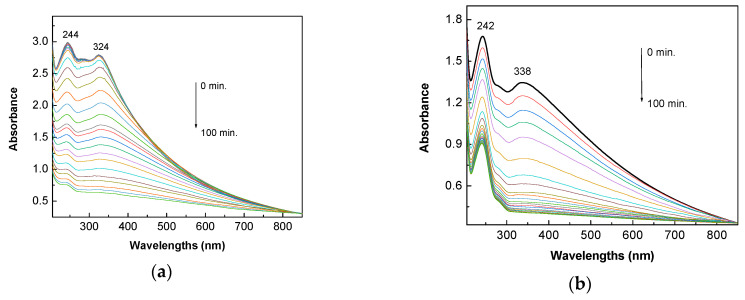

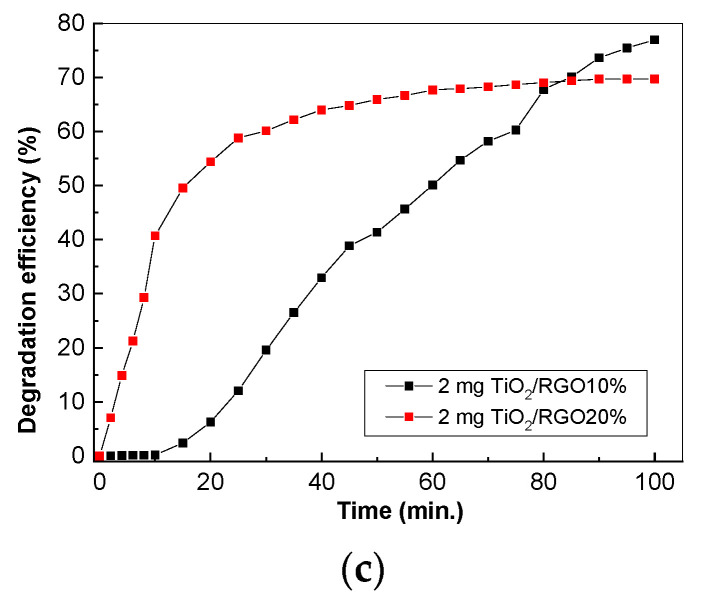
Evolution of UV-VIS spectra of 0.2 mM AC aqueous solution in the presence of 0.2 mg/mL TiO_2_/RGO blends with RGO concentrations equal to 10 wt. % (**a**) and 20 wt. % (**b**) when the samples are exposed to UV light for 100 min. The degradation efficiency of the AC aqueous solution (0.2 M) in the presence of 0.2 mg/mL TiO_2_/RGO blends with RGO sheet concentrations of 10 wt. % (black curve) and 20 wt. % (red curve) after subsequent exposure to UV light (**c**).

Figure 8 shows the influence of the concentration of RGO sheets in the TiO_2_/RGO blend. Before exposure to UV light, the ratio between the absorbance values of bands peaking at 240–244 nm and those at 338–320 nm is equal to ~1.1 (Figure 8a) and 1.2 (Figure 8b) when the concentration of RGO sheets in the TiO_2_/RGO blend is increased up to 10 wt. % and 20 wt. %, respectively.

After exposure to UV light, the photodegradation efficiency of the 0.2 mM AC aqueous solution with RGO concentrations equal to 10 wt. % and 20 wt. % in the TiO_2_/RGO blend (0.2 mg/mL) is equal to 76.95% and 69.69%, respectively (Figure 8c).

**Figure 9:**
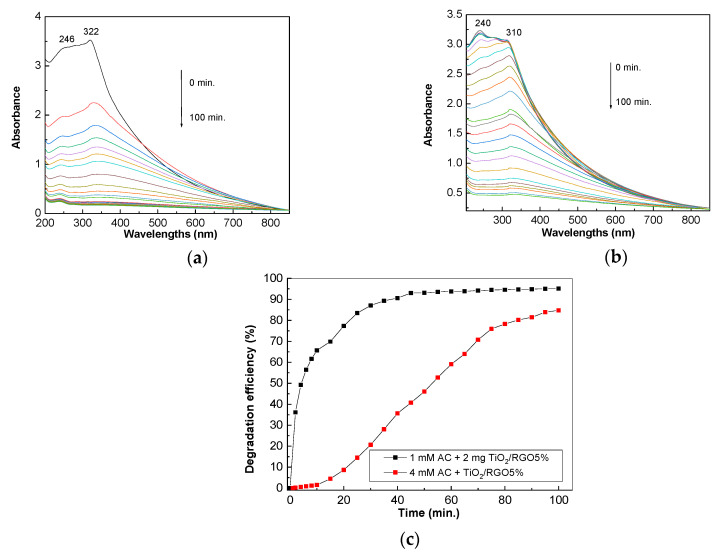
Evolution of the UV-VIS spectra of the AC in the presence of 0.2 mg/mL TiO_2_/RGO blends with RGO concentrations of 5 wt. %, with AC concentrations of 0.1 mM (**a**) and 0.4 mM (**b**). Degradation efficiency of AC in the presence of 0.2 mg/mL TiO_2_/RGO blends with an RGO concentration of 5 wt. %, when the blends were dispersed in 0.1 mM (black curve) and 0.4 mM (red curve) AC aqueous solution after subsequent exposure to UV light (**c**).

Figure 9 shows the efficiency of TiO_2_/RGO blends with RGO concentrations of 5 wt. % in relation to an AC solution with concentrations of 0.1 mM and 0.4 mM. Before exposure to UV light, the increase in AC concentration from 0.1 mM (Figure 9a) to 0.4 mM (Figure 9b) induces a hypsochromic shift of the band assigned to the π–π* electronic transition from 322 nm (Figure 9a) to 310 nm (Figure 9b).

According to Figure 9c, the photodegradation efficiency of AC solutions with concentrations of 0.1 mM and 0.4 mM in the presence of 0.2 mg/mL of TiO_2_/RGO and with an RGO concentration of 5 wt. % is equal to 95.18% and 84.75%, respectively. The four-fold decrease in the AC solution’s concentration leads to an increase of 10.43%.

**Figure 10:**
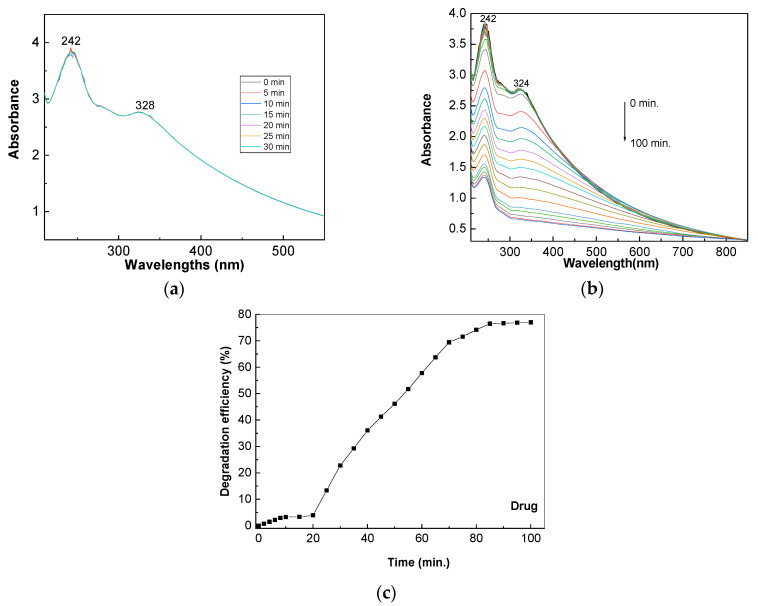
Evolution of the UV-VIS spectra of the paracetamol drug (0.2 mM) in the presence of 0.2 mg/mL TiO_2_/RGO blends with RGO concentrations equal to 5 wt. %, in the dark (**a**) and under UV light (**b**). Degradation efficiency of the paracetamol in the presence of 0.2 mg/mL TiO_2_/RGO blends with an RGO concentration of 5 wt. % dispersed in 0.2 mM paracetamol aqueous solution after subsequent exposure to UV light (**c**).

Figure 10 shows the evolution of the UV-VIS spectra of the TiO_2_/RGO blend (0.2 mg/mL) with an RGO concentration equal to 5 wt. % dispersed in a 0.2 mM AC solution containing excipients resulting from the dissolution of paracetamol in distilled water. When samples are in the dark for 30 min, no changes in the UV-VIS spectrum of the TiO_2_/RGO blend dispersed in 0.2 mM paracetamol aqueous solution occurred (Figure 10a). After exposure to UV light, a gradual decrease in the absorbance can be observed (Figure 10b). The photodegradation efficiency of paracetamol in the presence of a TiO_2_/RGO blend with an RGO concentration equal to 5 wt. % is 61.3% (Figure 10c). The lower AC photodegradation efficiency in the presence of the TiO_2_/RGO blend with an RGO concentration of 5 wt. % is related to the presence of the excipients of the paracetamol (Magistra C&C), namely, povidone, corn starch, croscarmellose sodium, and stearic acid.

### 2.3. Kinetic of the AC Photodegradation in the Presence of the TiO_2_/RGO Blends

The degradation rate constant of AC can be calculated using the equation:

$$\ln\left(\frac{A_{0}}{A_{t}}\right)=k\times t$$

where A_t_ and A_0_ are the absorbance values of the band peaking at 320–338 nm for the sample measured at t = 2–100 min and 0 min, respectively; k is the rate constant of the photodegradation reaction of AC at time (t).

**Figure 11:**
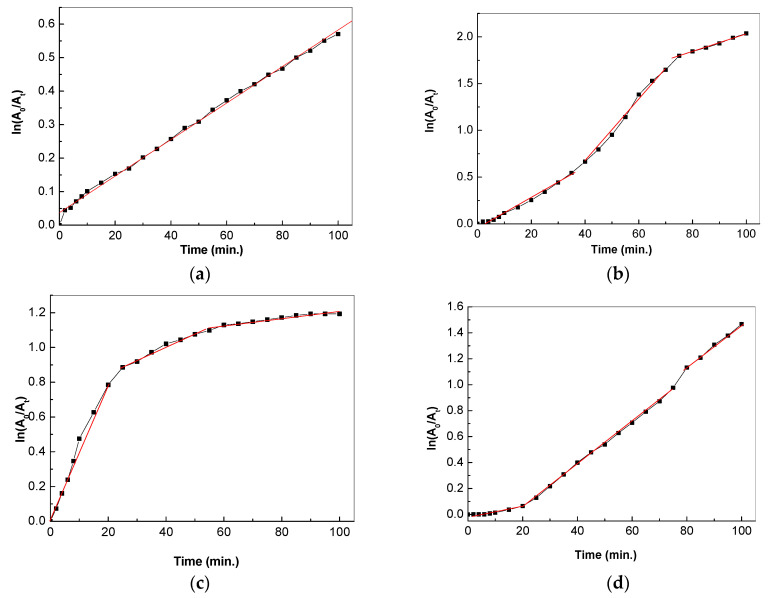
Reaction kinetics of the AC aqueous solutions (0.2 mM) in the presence of 0.05 mg/mL TiO_2_ (**a**) and 0.05 mg/mL TiO_2_/RGO with RGO concentrations of 5 wt. %, (**b**), 10 wt. % (**c**), and 20 wt. % (**d**) and UV light.

Figure 11a, showing the photodegradation of AC in the presence of TiO_2_ particles, adheres to a pseudo-first-order kinetic model. In the case of the TiO_2_/RGO blends, three linear regions are remarked in Figure 11b–d. The three linear regions correspond to: (i) the generation of the intermediate products caused by the photodegradation of the AC adsorbed onto the TiO_2_/RGO blend’s surface, with a rate constant of k_1_; (ii) the products resulting from ring cleavage [12] under UV light with a rate constant of k_2_; and (iii) the surface saturation process, with a rate constant of k_3_.

**Figure 12:**
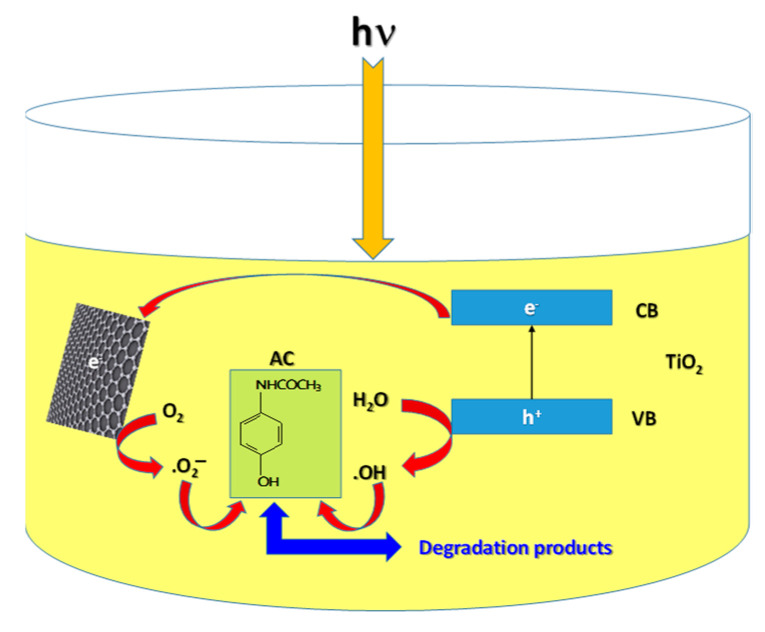
Mechanism of AC degradation in the presence of TiO_2_/RGO blends.

The reaction rate constant values for TiO_2_ and the three TiO_2_/RGO blends, as well as the corresponding linear regression coefficients for each stage, are shown in Table 1. According to Table 1, the rate constants show higher values in the cases of the TiO_2_/RGO blends with RGO concentrations equal to 5 wt. % and 10 wt. %, in comparison with TiO_2_. This suggests the higher loading of RGO sheets onto the TiO_2_ nanoparticles’ surfaces. The photocatalytic activity of the TiO_2_/RGO blends can be explained via mechanisms proposed by Moctezuma et al. [34] and Tao et al. [12]. Figure 12 shows the mechanism of AC degradation in the presence of TiO_2_/RGO blends.

**Figure 13:**
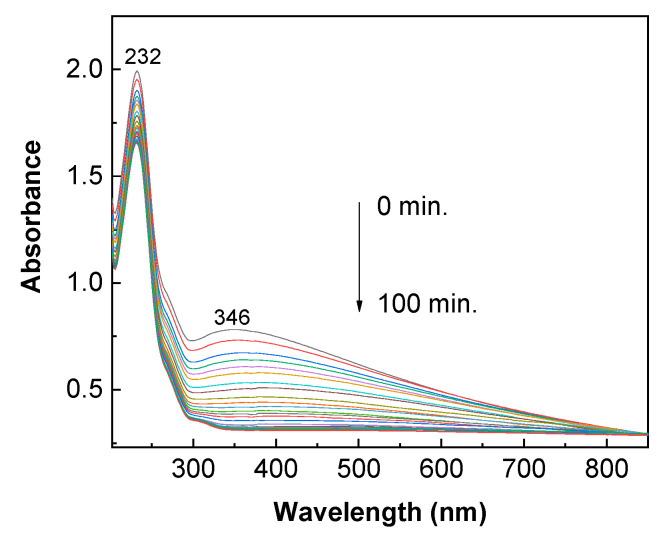
Evolution of the UV-VIS spectra of 0.2 mM AC solution in the presence of sample containing 0.2 mg/mL TiO_2_/RGO blend with an RGO concentration of 5 wt. % and IPA.

According to Figure 12, under UV light, holes are generated in the valence band (VB), and electrons appear in the conduction band (CB) of TiO_2_. Electrons from the CB of TiO_2_ are transferred to the RGO sheets, which can then interact with the O_2_ molecules dissolved in the AC aqueous solution, leading to the production of reactive oxygen species [35]. The holes from the VB of TiO_2_ can interact with the H_2_O, resulting in hydroxyl radicals, which, as unstable species, react with the AC when degradation products are created [36]. The significant role played by the hydroxyl radicals is highlighted when using isopropyl alcohol (IPA) as the quencher in the TiO_2_/RGO blends occurs Figure 13 shows the UV-VIS spectra of 0.2 mM AC solution in the presence of 0.2 mg/mL TiO_2_/RGO blend with a concentration of RGO of 5 wt. % and IPA. The exposure of this sample to UV light induces a decrease in the absorbance of the band peaking at 346 nm as the irradiation time is increased from 0 to 100 min. After 100 min of UV light exposure, the degradation efficiency reaches 60.25%, a value smaller than that reported in the case of the TiO_2_/RGO blend with a concentration of RGO of 5 wt. %–86.95% (blue curve in Figure 3e).

### 2.4. The Vibrational Properties of the TiO_2_/RGO Blend after AC Photodegradation

**Figure 14:**
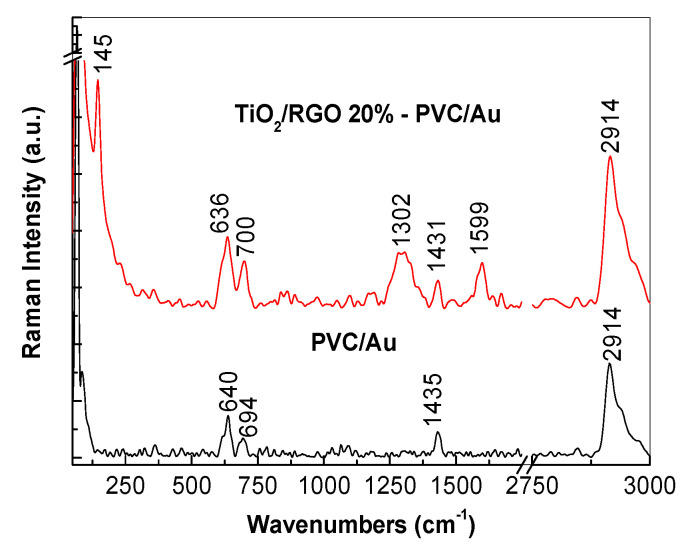
Raman spectrum of the PVC membrane modified with Au nanoparticles and a TiO_2_/RGO blend with a concentration of RGO of 20 wt.%, recovered after the photodegradation of a 0.2 mM AC solution and adsorbed onto the PVC membrane modified with Au nanoparticles.

Figure 14 shows the SERS spectra of the TiO_2_/RGO blend with a concentration of RGO of 20 wt. % after the photodegradation of a 0.2 mM AC aqueous solution.

The Raman spectra of the PVC membrane modified with Au nanoparticles are characterized by lines peaking at 640–694, 1435, and 2914 cm^−1^, assigned to the vibrational modes of C-Cl stretching, C-H symmetrical stretching in the CH_2_ group, and C-H asymmetrical stretching in the CH_2_ group, respectively [37,38,39,40]. In the case of the PVC membrane modified with Au nanoparticles adsorbed onto the TiO_2_/RGO blend derived from the photodegraded AC solution, the Raman spectra show: i) the three lines that belong to PVC peak at 636–700, 1431, and 2914 cm^−1^; and ii) the other three lines peak at 145, 1302 and 1599 cm^−1^, the first of which belongs to the E_g_ vibrational mode in TiO_2_ A, and the latter two of which are assigned to the breathing vibrational mode of the carbon hexagonal rings and the E_2_g phonon mode of RGO [29]. A careful analysis of the red curves in Figure 1 and Figure 14, i.e., of the Raman lines belonging to the constituents of the TiO_2_/RGO blend with an RGO concentration of 20 wt. %, before and after AC photodegradation, shows no changes. This indicates that no changes occurred in the vibrational properties of the TiO_2_/RGO blend during the AC photodegradation and that the catalyst can be used again in the pollutant removal process. In light of the results shown in this section, we note that the Raman lines of the TiO_2_/RGO blend were observed only in the case of PVC membranes modified with Au nanoparticles. The absence of the Raman lines of the TiO_2_/RGO blend in the case of the PVC membranes that were not modified with Au nanoparticles can be explained as a consequence of the low concentration of the TiO_2_/RGO blend (0.2 mg/mL) in the AC aqueous solution. In the present work, we overcame this issue by modifying the PVC membrane with Au nanoparticles, following the generation of surface plasmons (SPs) at the interface of the Au nanoparticles and the TiO_2_/RGO blend. As is well known, SPs have a significant role in the electromagnetic mechanism of the surface-enhanced Raman scattering (SERS) process, which explains the increase in the intensity of Raman spectra of various compounds. Considering the results shown in Figure 14, we can conclude that PVC membranes modified with Au nanoparticles can be used both as filters in wastewater management and as SERS supports.

### 2.5. The Stability of the TiO_2_/RGO Blends

**Figure 15:**
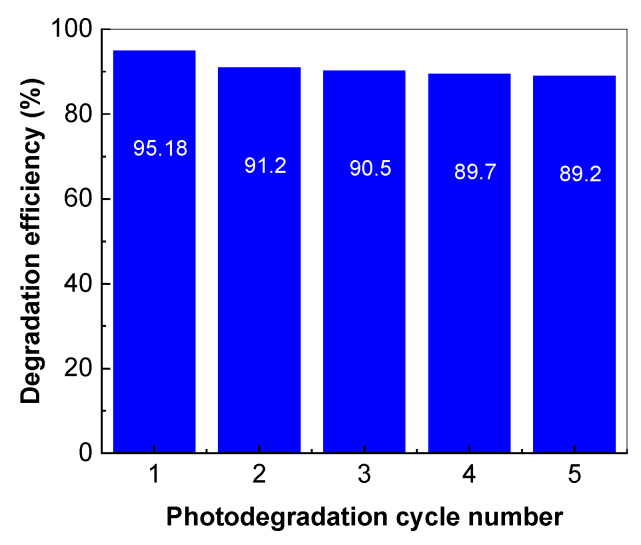
Variation in the degradation efficiency of 0.2 mM AC aqueous solution following the reuse of 0.2 mg/mL TiO_2_/RGO blend with a concentration of RGO equal to 5 wt. %.

Figure 15 shows the variation in the degradation efficiency of the 0.2 mM AC aqueous solution in the presence of 0.2 mg/mL TiO_2_/RGO blend with a concentration of RGO of 5%. After the first cycle of AC photodegradation, the solution was centrifuged to separate the TiO_2_/RGO blend from the aqueous solution. Subsequently, the TiO_2_/RGO blend was redispersed in distilled water and then dried at 80 °C in a vacuum for 30 min. This protocol was also carried out after each AC photodegradation cycle.

**Figure 16:**
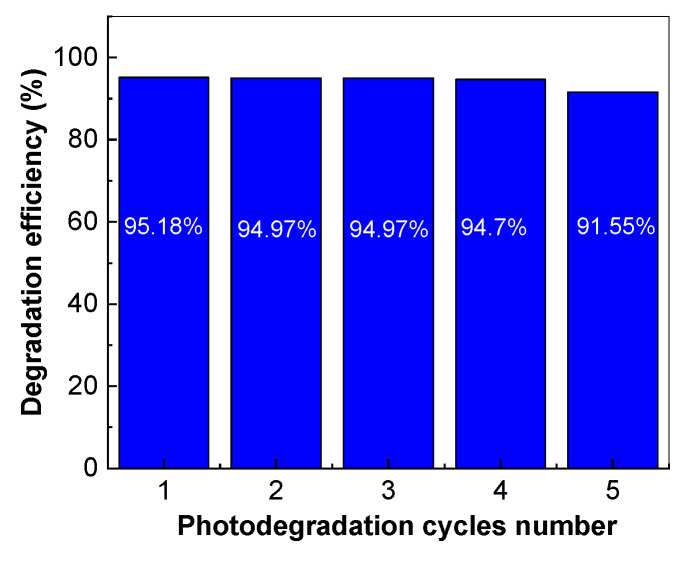
Variation in the degradation efficiency of 0.2 mM AC aqueous solution following the reuse of 0.2 mg/mL TiO_2_/RGO blend with a concentration of RGO equal to 5 wt. %.

According to Figure 15, when using the TiO_2_/RGO blend with a concentration of RGO of 5 wt.% in the five photodegradation cycles, a decrease in the degradation efficiency of AC from 95.18% to 89.2% was reported. No variation in the Raman spectra of the TiO_2_/RGO blend was observed after each photodegradation cycle of the AC solution. In the context of the photocatalytic degradation of AC in the presence of the catalysts based on TiO_2_ and RGO, the best results were published in Ref. [14] when a photodegradation efficiency of 100% was reported when the concentrations of AC and catalyst were equal to 0.05 mg/mL and 2 mg/mL, respectively. In our case, the photodegradation efficiency of AC in the presence of TiO_2_/RGO blend with a concentration of RGO equal to 5 wt.% was 95.18% when the concentrations of AC and catalyst were equal to 0.2 mM (equivalent to 0.03 mg/mL) and 0.2 mg/mL, respectively. An important advantage of the TiO_2_/RGO blends, prepared by the solid-state interaction, is their higher stability. The studies previously reported by A.H.C. Khavar et al. have highlighted that the reuse of the catalyst led to a 12% decrease in efficiency after five successive photodegradation cycles of AC [14]. In the present work, the photodegradation efficiency of AC by reusing the catalyst after five successive photodegradation cycles was only 5.98% (Figure 15). The drying of the catalyst at a temperature of 100 °C in air, time of 30 min, leads to a decrease in the photodegradation efficiency from 95.18% to 91.55%, i.e., only 3.63% (Figure 16).

This fact allows us to conclude that the TiO_2_/RGO blends prepared in this work show suitable stability in the photodegradation processes of AC.

**Figure 17:**
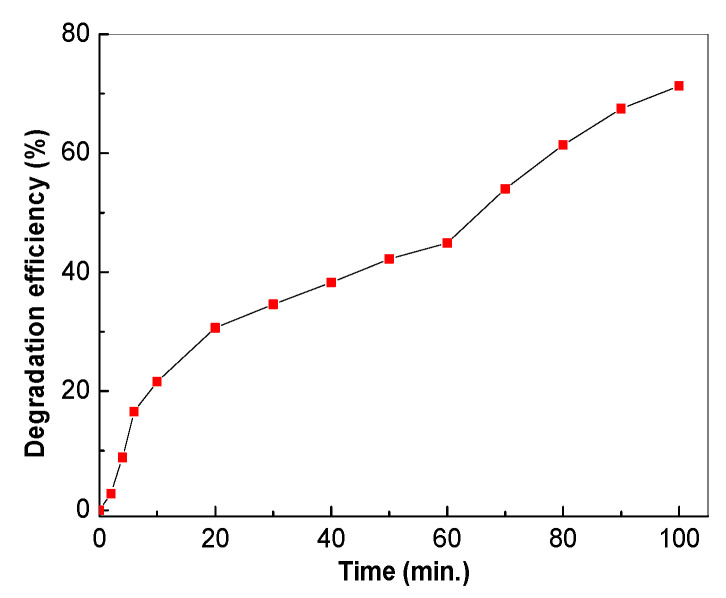
Degradation efficiency of 2 mg/mL AC in mineral spring water in the presence of 0.2 mg/mL TiO_2_/RGO blends with an RGO concentration of 5 wt. % after subsequent exposure to UV light.

In order to highlight the performance of the catalyst in real conditions, the testing of the TiO_2_/RGO blend in the mineral spring water containing 2 mg/mL AC was carried out. Using the TiO_2_/RGO blend with a concentration of RGO equal to 5 wt. %, the degradation efficiency of 2 mg/mL AC in mineral spring water was only 71.29% (Figure 17).

In this stage of our studies, it is significant to note that, depending on the type of wastewater, various technologies for the treatment of contaminants are used [41], and photodegradation processes are often influenced by the chemical composition of wastewater [42]. The chemical composition of the mineral spring water used in this work was as follows: 2.88 mg/L Na^+^, 1.18 mg/L K^+^, 2.63 mg/L Mg^2+^, 9.53 mg/l Ca^2+^, 0.03 mg/L SO_4_^2−^, 48.8 mg/L HCO_3_^−^, 74 mg/L NO_3_^−^, and 0.01 mg/L NO_2_, the sample having pH equal to 7.04. We are tempted to explain the decrease in the photodegradation efficiency of AC to be induced by the ions SO_4_^2−^, NO_3_^−^, Na^+^, and K^+^, which have been reported as agents that hamper the degradation of organic pollutants [42].

## 3. Materials and Methods

The TiO_2_ particles, PVC, IPA, and tetrahydrofuran (THF) were purchased from Sigma-Aldrich (St. Louis, MO, USA) as raw materials with a purity of 99.99%. According to our previous study, TiO_2_ particles contain both anatase and rutile crystalline phases [43]. RGO sheets were prepared from graphene oxide sheets and synthesized according to the protocol published by D.C. Marcano et al. [44], which were then interacted with hydrazine [29].

The TiO_2_/RGO blends were prepared by the solid-state interaction of the commercial TiO_2_ particles with RGO sheets by grinding the two compounds for 15 min until a change in the color of the TiO_2_ powder from white to gray was observed. Three TiO_2_/RGO blends with RGO concentration of 5 wt. %, 10 wt. %, and 20 wt. % were prepared.

In order to highlight the vibrational properties of the catalyst after AC photodegradation, a TiO_2_/RGO blend with 20% RGO was vacuum-filtered using a poly(vinyl chloride) (PVC) membrane with a diameter of 1 cm, on the surface of which 0.5 mL Au nanoparticles with a size of 10 nm were sprayed. The PVC membrane was prepared by the phase inversion method [45]. Briefly, 0.4 g PVC grains were dissolved in 10 mL THF under ultrasonication. The resulting solution was poured into a Petri vessel in order to carry out a thermal treatment at a temperature of 100 °C for 1 h to enable DMF evaporation. After this, the PVC membrane was removed from the Petri vessel and immersed in a water bath in order to remove the residual DMF. Finally, the PVC membrane was dried to a constant mass with a thickness of 40 μm. The PVC membrane modified with Au nanoparticles was used for the vacuum filtration of the TiO_2_/RGO blend after the photodegradation of the AC solution and the drying of the membrane to a constant mass. The modification of the PVC membrane with Au nanoparticles was achieved by spraying 0.5 mL of Au nanoparticles onto the PVC membrane’s surface and then performing an annealing treatment at 100 °C for 20 min in order to fix the Au nanoparticles onto the PVC membrane. Using the method published by Piella et al. [46], Au nanoparticles of 10 nm were synthesized. The modification of PVC membranes with Au nanoparticles was undertaken such that these membranes could be used as supports for surface-enhanced Raman scattering (SERS) spectroscopy.

In order to assess the photocatalytic properties of the four TiO_2_/RGO blends used in the photodegradation of AC, colloidal dispersions were prepared by adding each blend to an aqueous solution of AC followed by magnetic stirring for 20 min at 25 ^o^C in the dark, until reaching the adsorption–desorption equilibrium of AC on the surfaces of the TiO_2_/RGO blend catalysts. All colloidal dispersions were subsequently irradiated with UV light for 100 min with a halogen lamp of 100 W.

The Raman spectra of the three TiO_2_/RGO blends and TiO_2_ particles were recorded with a MultiRam FT-Raman spectrophotometer from Bruker (Bruker Optik GmbH, Ettlingen, Germany), in the backscattering geometry, with a resolution of 1 cm^−1^. The characterization of the TiO_2_/RGO blend with 20 wt. % RGO, after its use for AC removal, was also performed by Raman scattering.

The IR spectra of the three TiO_2_/RGO blends and TiO_2_ particles were recorded with a Vertex 80 FTIR spectrophotometer from Bruker (Billerica, MA, USA), with a resolution of 2 cm^−1^.

XPS spectra of the RGO sheets, TiO_2_ particles, and TiO_2_/RGO blends were recorded with a SPECS spectrometer endowed with an Al Kα source and a PHOBIOS 150 analyzer.

SEM and EDS analyses of the TiO_2_ particles, RGO sheets, and TiO_2_/RGO blends were undertaken with a Zeiss Gemini 500 field-emission scanning electron microscope with a Bruker EDS detector attached.

UV-VIS spectra of the solutions of AC containing TiO_2_/RGO blend catalysts, and TiO_2_ particles were recorded with a Lambda 950 UV-VIS-NIR spectrophotometer from Perkin Elmer (PerkinElmer, Inc., Waltham, MA, USA), with a resolution in the UV-VIS range of 0.05 nm.

## 4. Conclusions

In this work, we have reported novel results concerning the photocatalytic properties of TiO_2_/RGO blends prepared by the solid-state interaction of their two constituents in relation to AC degradation. Using IR spectroscopy and Raman scattering, we have demonstrated that with increases in the RGO concentration in the TiO_2_/RGO blend, the following occurs: a) A shift in the IR band, assigned to the Ti–O–Ti vibrational mode, from 692 cm^−1^ to 698 cm^−1^, simultaneously with an increase in the absorbance of the IR band peaking at 3627–3728 cm^−1^, which is assigned to the stretching vibrational mode of the OH bond. The increase in absorbance of the IR bands peaking at 1737 and 3627–3728 cm^−1^ indicates preferential adsorption of TiO_2_ particles onto the RGO sheets’ surfaces via the water molecules on the TiO_2_ particles’ surfaces. b) A decrease in the Raman line peaking at 147 cm^−1^, attributed to the E_g_ mode in TiO_2_ nanoparticles that show an anatase crystalline structure, the variation in which is accompanied by the presence of the D and G bands of the RGO sheets and the shift of the D band from 1292 cm^−1^ to 1301 cm^−1^. The best photocatalytic properties were obtained for the TiO_2_/RGO blend with an RGO concentration of 5 wt. %, when approx. 95.18% AC removal was achieved after 100 min of UV irradiation. The reaction kinetics of AC aqueous solutions in the presence of the TiO_2_/RGO blends with various RGO concentrations show a complex kinetic model of the pseudo-first-order, separated into three stages. The reuse of the TiO_2_/RGO blends after the first cycle of AC photodegradation indicates their suitable stability during all five cycles of the photodegradation of pharmaceutical compounds. In this paper, evidence is given regarding the use of PVC membranes modified with Au nanoparticles as filters for removing the catalyst from decontaminated water and as SERS supports when seeking to identify the vibrational properties of recovered and reused catalysts.

## Figures and Tables

**Table 1:** Reaction kinetics constants of the AC aqueous solutions in the presence of TiO_2_ and the TiO_2_/RGO blend with RGO concentrations of 5 wt. %, 10 wt. %, and 20 wt. %.

Sample Name	$k_{1}$ (min^−1^)	$R_{1}^{2}$	$k_{2}$ (min^−1^)	$R_{2}^{2}$	$k_{3}$ (min^−1^)	$R_{3}^{2}$
TiO_2_	0.005	0.9979				
TiO_2_/RGO 5%	0.016	0.9943	0.036	0.9959	0.0096	0.9982
TiO_2_/RGO 10%	0.040	0.9927	0.0073	0.9899	0.0052	0.9997
TiO_2_/RGO 20%	0.0052	0.9869	0.0161	0.9988	0.0168	0.9990

## Data Availability

The data are available upon request.

## Sample Availability

Samples of the compounds are available from the authors.
